# *The Organon* and Materia Medica Club of the Bay Cities of California

**Published:** 1896-11

**Authors:** W. E. Ledyard

**Affiliations:** Secretary


### *THE ORGANON* AND MATERIA MEDICA CLUB OF THE BAY CITIES OF CALIFORNIA.

The regular semi-monthly meeting was held at the office of Dr. George H. Martin, 606 Sutter Street, San Francisco, Friday evening, June 5th, 1896.

Members present: Drs. J. M. Selfridge, W. E. Ledyard, A. McNeil, George H. Martin, M. F. Underwood, C. M. Selfridge, and G. J. Augur.

The meeting was called to order at 8.30 o'clock by the President, Dr. J. M. Selfridge. The minutes of the previous meeting were read by the Secretary, and after corrections by Dr. McNeil, were approved. The Secretary then stated that he had received a letter from Dr. Yingling, saying that a society of "Purists" was about to be formed, and he wished the constitution and by-laws of *The Organon* Club, which had been forwarded to him.

Dr. McNeil then read a paper entitled "The Homœopathic Library," which was well appreciated by the members.

Dr. J. M. Selfridge read from *The Organon*, Section 142.

### Discussion.

Dr. McNeil—There is another form that has been used. Some symptoms cured have been given as clinical.

Dr. Underwood—I would like to know of a drug with the symptom, *constant wiggling of the toes during sleep.* I have a patient who has had it for twenty-five years.

Sections 143–148 were read.

DISCUSSION.

Dr. J. M. Selfridge—This is Hahnemann's explanation of how remedies cure.

Dr. Martin—It is the key-note of Homœopathy.

Dr. McNeil—There have been a great many explanations.

Dr. J. M. Selfridge—My explanation is, that the remedy being similar, it restores the disturbed molecular action.

Dr. Underwood—Disease is called a *distunement of the vital force*, which would be the effect of the extraneous cause.

Dr. McNeil—Chemistry may explain some things, but it cannot explain vital processes.

Dr. J. M. Selfridge—The atoms or molecules of the body are put into action by the life or "vital force." Any external cause, as severe cold, heat, etc., disturbs the molecular motion; then comes in the similar remedy and restores the normal molecular action. Life is a spiritual essence, and it is difficult to understand how a spiritual thing can be disturbed.

Dr. Underwood—Suppose a man gets sick from fright? You cannot disturb the molecules by fright.

Dr. J. M. Selfridge—The fright causes a contraction of the blood-vessels through the nerve centres.

Dr. Martin—The vital force is the same as the steam in an engine. Fright disturbs the molecular action, and not the vital force at all.

Dr. McNeil—Morbific potencies, as cold, heat, etc., distune the vital force. The similar remedy comes in and restores it.

Dr. J. M. Selfridge—Take as an illustration tuning-forks of different pitch, differently constructed, etc. There will be different sounds. Vibrations are sound waves. If you strike one of them the vibrations will seek out the particular tuning-fork that is molecularly like itself. Heat, cold, etc., do not disturb anything but the molecules of the particular part affected.

Dr. Martin—The body of ours is a machine, and the vital force sets it in motion. It is no more a part of the body than the steam which sets an engine in motion.

Dr. Underwood—Steam is material, as the engine, cogs, etc., are. The mind, or vital force, is the first thing that is disturbed.

Dr. McNeil—In disease, the vital force is distuned and the molecules set in action.

The meeting was then declared adjourned, to meet again the third Friday in June, at the office of Dr. J. M. Selfridge, in Oakland, when the reading of *The Organon* would be commenced at Section 149.

W. E. LEDYARD, *Secretary*.

Reported by Eleanor F. Martin, M. D.

